# Identification of CtE1 gene nucleotide polymorphisms and development of SNP-based KASP markers in guar (Cyamopsis tetragonoloba (L.) Taub.)

**DOI:** 10.18699/vjgb-25-134

**Published:** 2025-12

**Authors:** L. Criollo Delgado, D. Zewude, D.S. Karzhaev, D.E. Polev, E.К. Potokina

**Affiliations:** Skolkovo Institute of Science and Technology (Skoltech), Moscow, Russia; Skolkovo Institute of Science and Technology (Skoltech), Moscow, Russia; Institute of Forest and Environmental Management, Saint-Petersburg State Forest Technical University, St. Petersburg, Russia; Laboratory of Metagenomics Research, Saint-Petersburg Pasteur Institute, St. Petersburg, Russia; Skolkovo Institute of Science and Technology (Skoltech), Moscow, Russia

**Keywords:** guar, photoperiod, flowering time, CtE1 gene, GT-seq genotyping, SNP, KASP markers, гуар, фотопериод, время цветения, ген CtE1, GT-seq генотипирование, SNP, KASP-маркеры

## Abstract

Guar (Cyamopsis tetragonoloba (L.) Taub), is an important short-day legume crop, whose cultivation is limited at high latitudes due its photoperiod sensitivity, that negatively impacts flowering and maturation of this industrial-oriented crop. In its close relative, soybean, the E1 gene has been highly associated with the regulation of flowering time under long-day conditions. In this study we investigated the natural diversity of the E1 homologue gene (CtE1) in a panel of 144 guar accessions. For this purpose, the CtE1 gene was amplified and sequenced using Illumina. As a result, five novel SNPs were identified in the 5’-untranslated region, coding region, and 3’-untranslated region of the CtE1 gene. One non-synonymous SNP was located in the coding region causing a conservative Arg→Lys substitution. Based on the identified SNP, five KASP markers linked to polymorphism in the target gene were developed and tested in the guar collection. No significant associations were detected between discovered SNPs and available data on variability in flowering time or vegetation period length in the cohort of 144 accessions. These findings suggest that natural variation of the CtE1 gene in the studied germplasm collection has minimal effect on flowering or maturation. The limited functional allelic diversity observed in the CtE1 gene of guar compared to the E1 gene in soybean likely reflects differences in their evolutionary histories, domestication bottlenecks, and selection pressures.

## Abstract (ru)

Гуар (Cyamopsis tetragonoloba (L.) Taub) – бобовое растение короткого дня, возделывание которого в высоких широтах ограничено из-за его чувствительности к длинному фотопериоду, что негативно влияет на цветение и созревание этой новой индустриально значимой сельскохозяйственной культуры. У ее близкого родственника – сои – ген E1 вовлечен в регуляцию процессов цветения в условиях длинного дня. В нашем исследовании естественное разнообразие гена CtE1 (гомолога E1) проанализировано на выборке из 144 образцов гуара. Для этой цели ген CtE1 был амплифицирован и секвенирован с использованием Illumina, в результате чего идентифицировано пять новых SNP в 5’- и 3’-нетранслируемых областях, а также в кодирующей части гена CtE1. На основе выявленных SNP разработаны и протестированы пять KASP-маркеров, связанных с полиморфизмом целевого гена в изученной коллекции гуара. Один несинонимичный SNP был локализован в кодирующей области; этот полиморфизм приводит к консервативной аминокислотной замене Arg→Lys в кодируемом белке. Значимой связи между обнаруженными SNP и имеющимися данными об изменчивости сроков начала цветения или продолжительности вегетационного периода в выборке из 144 образцов не обнаружено. Полученные результаты свидетельствуют о том, что естественный нуклеотидный полиморфизм гена CtE1, представленный в изученной коллекции образцов гуара, не оказывает существенного влияния на цветение и созревание растений. Ограниченное функциональное аллельное разнообразие, выявленное в гене CtE1 гуара по сравнению с аллельным разнообразием гена E1 у сои, вероятно, отражает различия в их эволюционной истории и различные направления искусственного отбора в процессе селекции

## Introduction

Guar (Cyamopsis tetragonoloba (L.) Taub), is an industrialoriented short-day legume crop mainly cultivated for the production of guar gum (galactomannan) – a compound present in the seed endosperm of guar. This polysaccharide forms a viscous gel in water, and due to its thickening properties is widely used in several industrial sectors including oil and gas industry, cosmetics and food production (Benakanahalli et al., 2021). Currently, India and Pakistan are the main manufacturers and exporters of guar gum in the world market. However, there is growing interest in guar gum in many countries, and in the past two decades, guar rightfully gained the status of an important economical crop worldwide (Verma et al., 2025)

The main limiting factor for guar cultivation in Russia is its photoperiod sensitivity, which affects the timing of flowering and maturation of guar plants (Grigoreva et al., 2021 a, b). For the closely related legume soybean, loci that influence flowering and maturation under long-day conditions have been the subject of in-depth study for decades (Cao et al., 2017; Han et al., 2019). As a result, different alleles of genes involved in the photoperiod response were discovered, which are now used in breeding programs to adapt soybean varieties to diverse geographic regions and farming systems (Liu et al., 2020).

Among the genes identified to date as related to soybean vegetation period, E1 has been recognized as the most critical regulator of flowering time in soybean (Watanabe et al., 2012; Xia et al., 2012), and as a key selection locus in breeding programs (Xia, 2017). These characteristics made E1 the first and most significant target for CRISPR-Cas mutagenesis, aimed at developing new soybean germplasm with broad adaptability across different latitudes (Han et al., 2019).

Recently, an ortholog of the soybean E1 gene was identified in the guar genome, showing 80 % identity at the coding peptide level and a similar intron–exon structure (Criollo Delgado et al., 2025). Like the other members from E1 family genes, CtE1 encodes a protein containing a putative bipartite nuclear localization signal (NLS) and a DNA-binding B3-like domain. This suggests that the genetic pathways underlying the basic mechanisms of photoperiod response may be similar in soybean and guar, and therefore the selection of photoperiod-insensitive guar varieties may follow the same pathway as in soybean.

In soybean, the legume-specific E1 gene suppresses flowering of plants under long-day (LD) conditions, thus, nonsynonymous mutations in this gene result in a dysfunctional polypeptide, promoting flowering of plants in high latitudes (Xu et al., 2015). At least 5 misfunctional alleles were described for the E1 locus in soybean: e1-fs (frame shift), e1-as (amino acid substitution), e1-b3a (mutation in B3 domain), e1-re (retrotransposon insertion), e1-p (have SNPs or InDels in the coding sequence or 5′ upstream), and e1-nl (null) allele has a 130 kb deletion which includes the entire E1 gene (Liu et al., 2020). Development of functional markers for E1 polymorphisms has made significant contributions to both germplasm evaluation and marker-assisted selection (MAS) of soybean. Specifically, Kompetitive Allele Specific PCR (KASP) markers developed for SNPs at the E1-E4 loci, allowed to reveal the most advantageous allele combinations for soybean cultivars propagated in various regions of China (Liu et al., 2020). In this regard, it might be relevant to assess the level of polymorphism of the CtE1 gene in guar, represented in the natural intraspecific diversity of this legume crop, in order to identify alleles as possible targets for selection.

In the present paper we have evaluated nucleotide variability of CtE1 gene using the diversity panel of 144 guar accessions of different geographic origin. We developed KASP markers for all SNPs detected and estimated association between the revealed haplotypes and phenotypic performance of the guar varieties.

## Materials and methods

Plant material. A diversity panel consisting of 144 guar accessions, encompassing early- and late flowering/maturing varieties and landraces originating from India, Pakistan, United States, were described earlier (Grigoreva et al., 2021 a). In the same paper the performance of these accessions under field conditions in Krasnodar region (45°02′55″ N) in 2017 and 2018 was evaluated. Here, we used the field evaluation data to search for a link between alleles of CtE1 gene and variation of the agrobiological traits of guar plants from different accessions. Two traits most relevant to the putative function of CtE1 gene were considered: (1) flowering time defined as the number of days from sowing to flowering, recorded when 50 % of the plants in the accession have produced flower buds, and (2) length of vegetation period, which was calculated as the number of days from sowing to maturation (50 % of plants per accessions had mature pods).

Isolation of DNA, amplification of PCR and Sanger sequencing. As a first step, Sanger sequencing of CtE1 was performed on several plants with contrasting maturation times to assess the presence of polymorphisms within a small but diverse panel of genotypes. For Sanger sequencing genomic DNA from one plant per each accession was extracted from the 7-days seedlings following the protocol described by Ivanova et al. (2008). For further high-throughput genotyping using Illumina, a bulk DNA (5–7 plants) per accession was analyzed.

The extracted DNA was stored at −20 °C and subsequently assessed for quality and integrity using 1.5 % agarose gel electrophoresis. DNA concentration and purity were measured using a NanoDrop spectrophotometer (Desjardins, Conklin, 2010). For Sanger sequencing genomic DNA was subjected to PCR using a pair of primers designed for the CtE1 predicted sequence (Criollo Delgado et al., 2025) to amplify the 5′ untranslated region (5′ UTR), coding region, and 3′ untranslated region (3′ UTR). Information on the primer sequences is present on Table 1 (primers E1-F and E1-R).

**Table 1.**
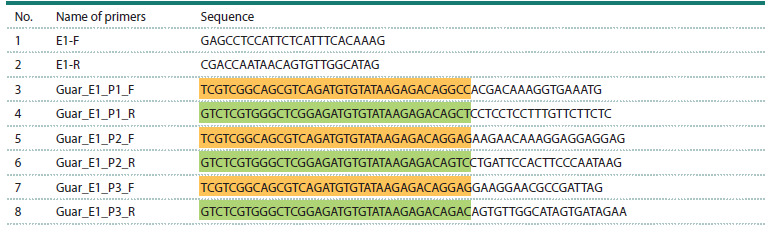
Primer pairs used for amplification of the CtE1 gene in guar. Primers 1, 2 were used for Sanger sequencing. Primers 3–8 were included for Illumina sequencing purposes; adapter sequences are marked in color: forward (yellow) and reverse (green)

PCR amplification was performed using primers E1-F and E1-R to generate 803 bp product. The PCR reaction mix (25 μL) consisted of 1 μl genomic DNA, 1× PCR buffer, 3 mM MgCl2, 0.4 μM of each primer, 100 μM dNTPs, and 2.5 unit of TaqDNA polymerase. The initial denaturation was performed at 94 °C for 2 min; followed by 30 cycles of denaturation at 98 °C for 30 s, annealing at 54 °C for 1 min, and extension at 72 °C for 45 s; and with a final extension at 72 °C for 10 min. The PCR products were purified from PCR mix using QIAquick PCR Purification Kit (Qiagen). The purified samples were submitted to a commercial sequencing facility (Evrogen, Moscow) for further processing

Primer design for Illumina sequencing. High-quality DNA samples of 144 guar accessions were used for PCR with specifically designed primers. Three pairs of primers were designed to amplify three overlapping regions of CtE1 gene covering the same region as for Sanger sequencing, but overhangs were included in the primer’s sequences to facilitate subsequent Illumina application. Table 1 lists the forward (Guar_E1_P1_F, Guar_E1_P2_F, Guar_E1_P3_F) and reverse (Guar_E1_P1_R, Guar_E1_P2_R, Guar_E1_P3_R) primer sequences, the overhangs are color-coded: yellow for the forward and green for the reverse primers. Primer design was performed using the Integrated DNA Technologies (IDT) online primer design tool (https://eu.idtdna.com/pages/tools/ primerquest).

PCR amplification was carried out using the cycler C1000 Touch (Bio-Rad, USA) to target the genomic region of CtE1 in the guar genome. Each primer pair was tested separately using genomic DNA as the template. The PCR mix (25 μL) contained 1× HF buffer, 0.4 μM of each primer, 200 μM dNTPs, and 1 unit of Phusion® High-Fidelity DNA Polymerase (NEB, USA). The thermal cycling conditions were as follows: initial denaturation at 98 °C for 2 min, followed by 30 cycles of denaturation at 98 °C for 30 s, annealing at 62 °C for 1 min, and extension at 72 °C for 45 s. A final extension step was performed at 72 °C for 10 min.

Library preparation, sample pooling and Illumina sequencing. The sequencing library was prepared for a Genotyping- in-Thousands by Sequencing (GT-SEQ) (Campbell et al., 2015) approach from the PCR-amplified products. All PCR products were first purified using ammonium acetate precipitation to eliminate unincorporated nucleotides, salts, and other impurities from the reaction mixture. The concentration of each cleaned DNA sample was then measured using a NanoDrop spectrophotometer (Desjardins, Conklin, 2010). Each sample had an initial volume of 20 μL. For pooling, 4 μL was taken from samples with a DNA concentration below 10 ng/ μL, while 2 μL was taken from samples with a concentration above 10 ng/μL. Approximately equal concentrations were allowed, since Illumina sequencing provides excess coverage of the target locus for each sample. This compensates for variability in concentration without exact quantification, which is acceptable for amplicon sequencing at high coverage depths (e. g., 16S) (Kennedy et al., 2014).

The selected volumes were pooled together into a single 2 mL Eppendorf tube to create a composite library. The pooled library was then prepared for high-throughput sequencing with the Illumina MiSeq. Nextera XT DNA Library Prep Kit was used for a two-step PCR workflow. First PCR was performed with gene specific primers + overhangs (Table 1), the second PCR was performed to add Illumina adapters + indices. For the pooled library, only one dual Illumina index was used, which significantly reduced the cost of sequencing. Pairedend sequencing was employed with 2 × 250 bp read mode.

Bioinformatics pipeline for SNP detection. Quality assessment of the raw reads was performed using FastQC (Andrews, 2010) with default parameters to evaluate base quality, GC content, and potential adapter contamination. Subsequently, high-quality reads were aligned to the reference target sequence, specifically, the CtE1 guar gene (Criollo Delgado et al., 2025), using the BWA-MEM algorithm. SNPs were then identified using a variant calling pipeline that involved SAMtools for alignment processing and BCFtools for SNP calling and variant filtration (Li, 2011; Danecek et al., 2021).

Development of KASP assays. The KASP primers genotyping assay design tool (https://primerdigital.com/tools/kasp. html) (Kalendar et al., 2022) was used to design KASP primers for detected SNPs. Two allele-specific primers were designed carrying unique tails: FAM (5′ GAAGGTGACCAAGTTCAT GCT 3′) and HEX (5′ GAAGGTCGGAGTCAACGGATT 3′) respectively, with the targeted SNPs at the 3′end (penultimate nucleotide), and a common primer was designed to pair with both forward and reverse primers. KASP genotyping primers are provided in Table 2. SNP positions were numbered relative to the CtE1 coding sequence (CDS), where positive numbers indicate positions within the CDS (with 1 corresponding to the A of the ATG start codon), negative numbers (−) indicate nucleotides upstream (5′) of the start codon, and asterisks (*) denote nucleotides downstream (3′) of the stop codon (den Dunnen et al., 2016).

**Table 2.**
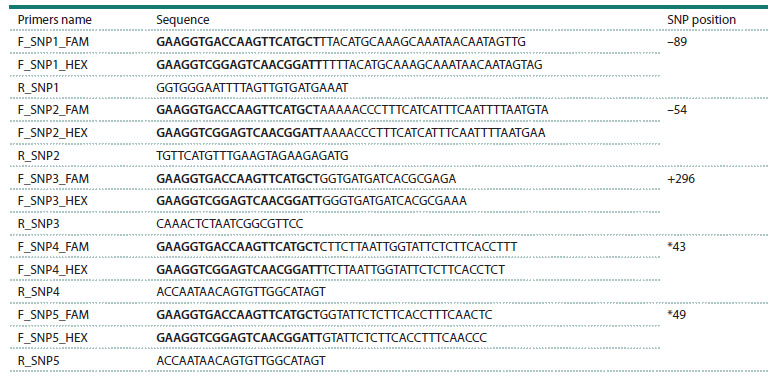
KASP primers for CtE1 gene assessment: two allele-specific forward primers with unique 5’ tails (shown in bold) and targeted SNPs at their 3’ ends

The KASP assay was conducted in 8 μL PCR reaction volume comprising 2 μL of genomic DNA (5 ng/μL), 3 μL of 2×KASP-TF V5.0 2X Master Mix (LGC, Biosearch Technologies) and 0.2 μL of allele-specific primer mix, making the final concentration of forward primers in the reaction volume 0.05 mM each, and 0.10 mM of common reverse primer. PCR cycling was performed with QuantStudio 5 cycler (Thermo Fisher Scientific, USA) using the following protocol: pre-incubation 30 °С 30 s (Pre-Read stage fluorescence measurement), pre-denaturation at 95 for 10 min, followed by 10 touchdown cycles (95 ℃ for 15 s; touchdown from 62 ℃ to 55 ℃ with 1.5 ℃ decrease per cycle for 60 s), followed by 60 additional cycles (94 ℃ for 20 s; 55 ℃ for 60 s), 30 °С for 1 min (Post-Read stage fluorescence measurement).

Statistical analysis. Descriptive statistics and estimates of variance were done by using the R package ‘agricolae’ (https://cran.r-project.org/package = agricolae) (de Mendiburu, 2023). To check the effect of allelic variants on flowering and maturation time traits, ANOVA was used. For each analysis of variance, we also evaluated normality of residuals distribution using the Shapiro–Wilk Test. When the assumptions for residuals normality were not satisfied, the Kruskal– Wallis rank sum test served as a robust non-parametric alternative.

## Results

**Identification of novel SNPs in the gene CtE1**

**Fig. 1.**
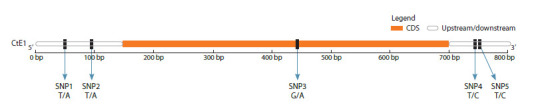
Location of SNPs in the CDS and upstream/downstream regions of the CtE1 gene revealed by Sanger sequencing among 8 guar cultivars differing in flowering/maturing time.

Sanger sequencing of CtE1 in eight guar varieties with contrasting maturation times confirmed the presence of polymorphisms in the sequence of the gene previously predicted in silico, and identified five SNPs (Table 3). Positions of the SNPs were determined relatively to the CDS of the CtE1 gene (Criollo Delgado et al., 2025) and a reference sequence of 1846 bp encompassing the CDS and upstream/downstream regions of the CtE1 gene, which was extracted from guar genome assembly Cte V1.0 (GCA_037177725.1). Two SNPs were identified in the upstream region of the CtE1 gene, the first SNP was located in the 5′ untranslated region (5′ UTR) at position –89 relative to CDS (SNP1) and the second SNP was located in the 5′ UTR at position –54 (SNP2), where the nucleotide thymine (T) was substituted with adenine (A) in both cases. One non-synonymous SNP was found within the coding region at position 296 from the start codon (SNP3), showing a guanine (G) to adenine (A) substitution that causes an amino acid change from arginine to lysine. Additionally, two SNPs were detected in the 3′ untranslated region (3′ UTR) at positions *43 (SNP4) and *49 (SNP5), both involving a transition from thymine (T) to cytosine (C). The analyzed CtE1 gene sequence spans 803 base pairs, covering the 5′ UTR, coding region, and 3′ UTR, and all 5 SNPs are shown in Figure 1. As shown in Table 3, out of the 8 varieties examined by Sanger sequencing, the only distinct CtE1 haplotype was revealed in accession Cat.52580.

**Table 3.**
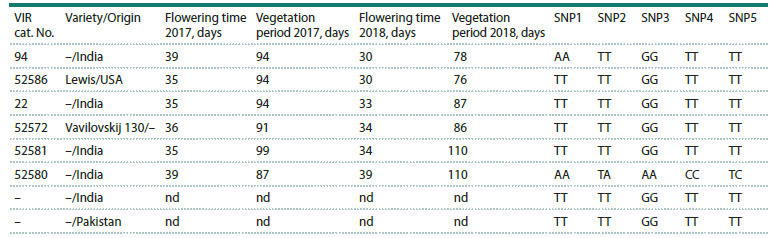
Polymorphisms of the CtE1 gene among 8 guar accessions showing variation of flowering and maturation time Note. For the accessions the variety/origin is indicated if known. nd – not determined

**Illumina sequencing**

**Fig. 2.**
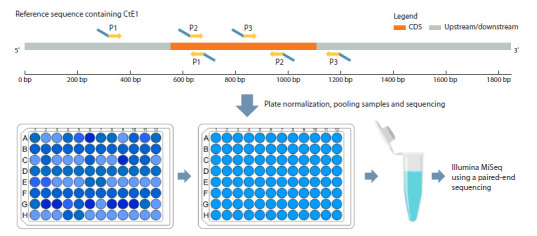
Scheme of the GT-seq approach and distribution of primers for GT-seq in the CDS and upstream/downstream regions of the CtE1 gene. By numbers primer pairs (forward and reverse) are indicated. The green part of the primers represents the target-specific sequence. Blue tails of the primers represent the Illumina sequencing adapters.

To extend the CtE1 genotyping to the entire guar collection of over 144 accessions we avoided the use of cost-consuming Sanger sequencing and instead applied a method previously

described as Illumina Genotyping in Thousands by Sequencing (GT-Seq) (Campbell et al., 2015). We created a pooled library containing multiplex PCR products of 3 regions spanning the CtE1 gene to identify all possible polymorphisms in the target sequence in the collection of 144 accessions. Three pairs of primers with Illumina sequencing adapters were designed (Table 1) enabling all amplicons of all individuals to be pooled into a single sequencing library. Figure 2 shows the location of the primers in the sequenced region (847 bp) compared to a reference sequence of 1846 bp.

No barcoding of individual samples was performed, so when running the Illumina MiSeq, only one dual Illumina index was used to barcode the entire library. As a result, 4,880,840 raw reads were obtained from Illumina and qualitychecked using FastQC to evaluate base quality, GC content, and adapter contamination. 4,513,800 (92.48 %) of the reads were then successfully aligned to the CtE1 reference guar genome assembly (Cte V1.0, GCA_037177725.1). With the data available, each of the three amplicons was covered by an average of 10,448 reads for each of the 144 guar accessions. As a result, the same 5 SNPs, that were discovered by Sanger sequencing of 8 accessions contrasting in flowering and maturing time, were again detected, and no additional polymorphism was found among 144 guar accessions

The GT-seq analysis revealed a single missense mutation in the coding sequence of the CtE1 gene within the examined intraspecific diversity of guar, resulting in an Arg→Lys amino acid substitution. However, unlike the loss-of-function mutations observed in soybean that lead to truncated or nonfunctional E1 proteins, no such deleterious variants were detected. Nevertheless, an attempt was made to assess the field performance of guar plants carrying different CtE1 alleles. To facilitate this, KASP assays were developed for the identified SNPs.

**High-throughput KASP genotyping of polymorphisms in the CtE1 gene**

**Fig. 3.**
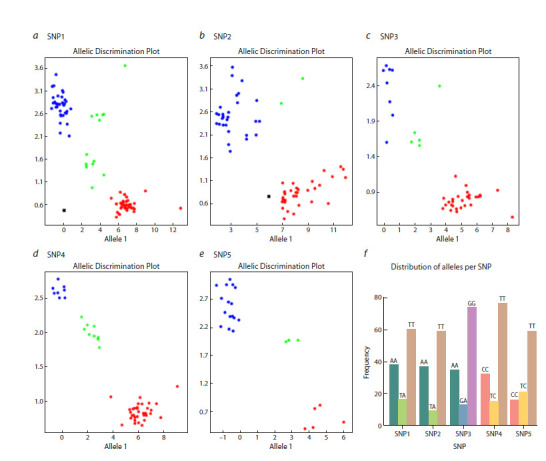
Clustering of alleles of SNPs in the CtE1 gene using KASP assays. а–e, allelic discrimination plots of KASP markers located on the five SNP loci. SNP1–SNP5 correspond to these in Table 3. The clusters of accessions are represented on the scatter plot on the x-axis (Allele 1) and y-axis (Allele 2); f, distribution of alleles per SNP loci. The Figure does not reflect genotyping results for the entire collection: if some samples were incorrectly or incompletely genotyped, they were re-analyzed in additional runs.

Five KASP markers linked to polymorphisms in the CtE1 gene were developed based on the SNPs identified through the Illumina GT-seq approach and tested with 144 guar accessions. Each KASP marker enabled apparent clustering of accessions into three genotype classes (homozygous allele1, homozygous allele2, and heterozygous) (Fig. 3). The heterozygosity level estimated for SNPs in the CtE1 gene in the studied collection of 144 guar accessions ranged from 0.086 to 0.218 (Fig. 3f ), which is in line with the average heterozygosity level of 0.127 reported for soybean germplasm collections (Potapova et al., 2023).

**Analysis of association between SNPs in the СtE1 gene and flowering/maturation time of guar varieties**

Since the guar diversity panel encompassing studied 144 guar accessions was evaluated in 2017 and 2018 in the field conditions of Krasnodar region (Grigoreva et al., 2021a), we use the opportunity to explore any possible correlation existing between the revealed nucleotide polymorphisms of CtE1 and agronomic performance of guar varieties carrying different alleles of the gene.

Flowering time observed for the studied accessions varied from 32 to 45 days in 2017 and from 29 to 42 days in 2018. Notably, the correlation between flowering time of the guar collection in 2017 and 2018 was not statistically significant (r2 = 0.05, p-value > 0.05). Heritability of flowering time in guar estimated under stable natural conditions in India ranged from 52 % (Remzeena, Anitha, 2021) to 81 % (Choyal et al., 2022). However, guar propagation in the Krasnodar region often faces challenges, such as, for example, the spring drought of 2018, which resulted in damage to plant seedlings. Therefore, the observed year-to-year variations in the flowering time of guar genotypes may be due to differences in environmental factors, as well as the lack of standardized agricultural practices for this recently introduced legume crop

Length of vegetation period varied respectively in the range 72–116 days and 72–110 days in two years. Here, the correlation between the overall vegetative period of guar accessions in 2017 and 2018 was low, but statistically significant (r2 = 0.19, p-value < 0.01). As with flowering time, this low correlation can presumably be explained by extreme drought conditions in spring 2018

Given genotyping data for 5 SNPs in the CtE1 gene for 144 guar accessions, we attempted to perform ANOVA for each SNP, considering the three genotypes (two alternative homozygous and heterozygous) as factor levels and the number of days from sowing to flowering (maturing) as the dependent variable.

Of all the markers we tested, only one SNP (SNP2) demonstrated an association with flowering time in guar in 2018, that approached statistical significance (ANOVA, p = 0.052). Heterozygous genotypes for SNP2 (TA) tended to flower slightly later than both homozygotes (TT and AA), a result that is difficult to explain from a biological perspective. Therefore, it can be concluded that the natural polymorphisms of the CtE1 gene identified in the available collection of 144 guar genotypes do not exert a significant effect on flowering or maturation time.

## Discussion

In the present study, we assessed natural allelic variation of the CtE1 gene using a diversity panel of 144 guar accessions from different geographic origins. CtE1 was previously identified as a homolog of E1, the major flowering time regulator in soybean; however, its genetic diversity and functional role in guar had not yet been reported. The E1 gene plays a key role in the functional network of photoperiodic flowering regulation in soybean. Since the molecular identity of this gene was successfully elucidated in 2012 (Xia et al., 2012), numerous studies have underscored the significant impact that mutations in this gene can have on photoperiod sensitivity (Zhai et al., 2015; Han et al., 2019; Fang et al., 2024a, b; Gao et al., 2024).

During the adaptation of cultivated soybean northward to high latitudes under longer daylengths, the E1 gene, like some other important flowering inhibitor genes (e. g., E3, E4, Tof5, Tof11, and Tof12), has accumulated sequence polymorphisms, which reduced photoperiod sensitivity to produce early flowering. Thus, the variation leading to early flowering was artificially selected, allowing cultivated soybean to adapt to high latitude areas (Lin et al., 2021). Several functional and non-functional/dysfunctional E1 alleles (e. g., E1, e1-as, e1-fs, e1-nl) have been identified in soybean, which vary by geography/ maturity group (Hou et al., 2023). However, not all of them equally contribute to flowering phenotype. For example, e1fs and e1nl are functionally deficient, leading to very early flowering and maturity, while e1-as is a weak mutant allele with an effect intermediate between that of the E1 genotype and the functionally deficient alleles (Xia et al., 2012).

The high similarity in coding peptide sequences between the soybean E1 gene and the guar CtE1 gene, along with their comparable intron-exon structures, suggests that the genetic pathways governing the fundamental mechanisms of photoperiod response may be conserved across these two legume species (Criollo Delgado et al., 2025). This structural and sequence conservation implies that intraspecific genetic variation at the CtE1 locus in guar could potentially contribute to variation in photoperiod sensitivity, similar to the functional allelic diversity observed at the E1 locus in soybean

However, within the natural allelic diversity of CtE1 evaluated in this study, no clearly dysfunctional alleles were identified. Of the five SNPs discovered in the CtE1 gene, only one (SNP3) was found in the coding region and resulted in a non-synonymous arginine-to-lysine substitution. This alteration is located at amino acid position 99, situated within the B3-like domain, which in guar spans amino acid residues 61–171 (Criollo Delgado et al., 2025). Among the soybean E1 polymorphisms discovered to date, a similar e1-b3a mutation was found, which also occurs in the middle of the B3 domain of the E1 gene. The e1-b3a represents 5bp (3 SNP and 2-bp deletion) mutation which leads to a frameshift causing a premature stop codon at the middle of the B3-like domain. As a result, the soybean e1-b3a/e1-b3a genotype flowered significantly earlier than E1/E1 and E1/e1-b3a (Zhai et al., 2015). In contrast, the functional significance of the Arg→Lys amino acid substitution identified within the B3 domain of guar, remains uncertain, as arginine and lysine share similar physicochemical properties. This substitution is considered conservative and is therefore predicted to have a minimal effect on protein function (Betts, Russell, 2003; Ryan, Ó’Fágáin, 2007; Banayan et al., 2024). On the other hand, it has been reported that amino acid substitutions in the E1 sequence also can lead to significant functional changes, if they occur in the region of bipartite Nuclear Localization Signal (NLS). For example, the point mutation from arginine to threonine at position 15 in the soybean E1 gene (known as e1-as mutant) occurs at exactly the first basic domain of the bipartite NLS, leading to different subcellular localization of the resulting protein and affecting flowering phenotype (Xia et al., 2012).

Two SNPs (SNP1 and SNP2) were discovered in 5′UTR region of the CtE1 gene. Similarly, mutations e1-re and e1-p were described at the 5′UTR region of the E1 gene in soybean. The e1-re allele is characterized by the insertion of a long interspersed nuclear element (LINE) located 148 bases upstream of the start codon, whereas the e1-p mutant exhibits sequence variation in the 5′ upstream region compared to E1. The effects of both alleles on flowering time in soybean have not been well studied (Tsubokura et al., 2014).

The limited functional allelic diversity observed in the CtE1 gene of guar, compared to the E1 gene in soybean, likely reflects differences in their evolutionary histories, domestication bottlenecks, and selection pressures. Soybean was domesticated in a region spanning 30–45°N in China and is now cultivated globally across a broad latitudinal range, from 53°N to 35°S (Lin et al., 2021). In contrast, guar was domesticated in India and Pakistan (Ravelombola et al., 2021), and to this day, these countries remain the primary centers of guar cultivation. This more geographically restricted domestication of guar and cultivation range may have resulted in reduced selection for photoperiodic adaptation and, consequently, lower allelic diversity at key flowering-time loci such as CtE1.

On other hand, it is still possible that within the intraspecific diversity of guar there exist genotypes carrying more severe mutations in the CtE1 gene that can substantially impair its function; however, such genotypes were not present in the studied cohort of 144 accessions.

Furthermore, it has been reported that E1 homologues in various legumes exhibit differing roles in flowering, highlighting functional diversification within the E1 gene family (Zhang et al., 2016; Cao et al., 2017). For instance, in Phaseolus vulgaris, the E1 homologue known as PvE1L acts as a flowering repressor, mirroring the function of the E1 gene in soybean. Ectopic expression of PvE1L has been shown to delay flowering onset in soybean (Zhang et al., 2016). In contrast, Medicago truncatula’s E1 homologue, MtE1L, does not influence flowering when ectopically expressed in soybean. This variation suggests that the functional roles of E1 homologues in legumes may be linked to lineage specificity and genomic duplication events. This underscores the complexity of flowering regulation within the legume family.

The CRISPR/Cas9 system has recently emerged as a powerful tool for targeted genome editing and functional genomics research. In soybean, its application has enabled in-depth investigation of the E1 gene’s role in photoperiod regulation, through CRISPR/Cas9-mediated mutagenesis followed by phenotypic analysis of flowering time (Wan et al., 2022). A similar approach can be implemented in guar by generating CRISPR/Cas9-induced mutants with targeted alterations in the CtE1 gene. This would allow for a direct functional assessment of CtE1 and its role in regulating flowering time and photoperiod sensitivity in guar. In addition, the application of CRISPR/Cas-based mutagenesis could potentially benefit guar breeding programs not only by enabling the creation of CtE1 mutants, but also by targeting other flowering-related genes homologous to the soybean E maturity genes, such as CtE2–CtE4. This approach could facilitate a detailed investigation of the genetic network regulating flowering time in guar. This may also facilitate the development of novel photoperiod- insensitive guar germplasm, analogous to soybean mutants that have expanded soybean cultivation into higher latitudes.

## Conclusion

In this study, we characterized nucleotide variability of the CtE1 gene, the guar ortholog of soybean E1 gene, in a diverse panel of 144 guar accessions and identified five novel SNPs across the 5′ UTR, coding, and 3′ UTR regions. We developed KASP markers for these SNPs to provide a robust genotyping tool to explore CtE1 haplotypes in larger germplasm collections. Genotyping of 144 guar samples for five CtE1 SNPs revealed only one SNP in the coding part of the gene, causing an Arg→Lys substitution. Given the conservative nature of this amino acid substitution, its functional impact is likely limited. No significant associations were detected so far between discovered SNPs and available data on variability in flowering time or vegetation period length. Our findings indicate that natural variation in CtE1 within the studied guar germplasm has little impact on flowering time or maturation. We hypothesize that the geographically restricted domestication and cultivation range of guar may have led to reduced selection pressure for photoperiodic adaptation, resulting in lower allelic diversity at key flowering-time loci such as CtE1

## Conflict of interest

The authors declare no conflict of interest.
